# Unusual bilateral lymphadenopathy revealing Hodgkin’s lymphoma: a case report

**DOI:** 10.1007/s12672-025-04371-5

**Published:** 2025-12-31

**Authors:** Vaishnavi D. Rajurkar, Keshao Hiwale, Sahitya Vodithala, Nisha D. Barole, Prajakta Nimbalkar

**Affiliations:** 1 Department of Medical Laboratory Technology, Datta Meghe Institute of Higher Education and Research, Sawangi, 442001 Wardha, Maharashtra India; 2 Department of Pathology, Datta Meghe Institute of Higher Education and Research, Sawangi, 442001 Wardha, Maharashtra India; 3 Department of Clinical Research, Datta Meghe Institute of Higher Education and Research, Sawangi, 442001 Wardha, Maharashtra India

**Keywords:** Hodgkin’s lymphoma, Reed-sternberg cells, Bilateral lymphadenopathy, Differential diagnosis, Tuberculosis

## Abstract

Hodgkin lymphoma is a rare lymph node malignancy that has the presence of Reed-Sternberg cells. We describe a case of a 72-year-old male with progressive bilateral cervical and inguinal lymphadenopathy with the absence of constitutional symptoms. Primary clinical and radiographic assessment was in favor of an infectious or metastatic etiology. Classical Hodgkin lymphoma was confirmed by excisional biopsy of the lymph nodes that demonstrated classic Reed-Sternberg cells in a polymorphous inflammatory background. Additional support to the diagnosis was provided by immunohistochemistry. The patient was staged at Ann Arbor stage IIIB disease and sent to oncologic care, where he was placed on systemic chemotherapy. The case demonstrates that persistent lymphadenopathy can be a hard-to-detect disease, particularly in tuberculosis-endemic regions, and that a timely biopsy and comprehensive evaluation are necessary to avoid diagnostic lag time and offer treatment before it is too late.

## Introduction

Hodgkin’s lymphoma (HL) stands as a unique malignancy that affects less than 1% of all cancers worldwide and constitutes 10% of lymphomas. Medical experts can identify this disease through microscopic examination of Reed-Sternberg cells surrounded by reactive inflammatory cells [1]. The typical cancer pattern of Hodgkin’s lymphoma affects one group of lymph nodes, while late-stage presentations tend to spread throughout multiple areas. Among all affected lymph node locations, the cervical nodes show the highest frequency, while mediastinal and axillary nodes follow as secondary sites [2]. The simultaneous appearance of lymphadenopathy across both cervical and inguinal regions remains an uncommon presentation of HL that medical professionals must differentiate from infections, including tuberculosis and metastatic cancers, particularly among people living in areas where granulomatous diseases are prevalent [3]. This case stands out because it shows both cervical and inguinal lymphadenopathy in addition to “B” symptoms such as fever, weight loss, and skin itch, which expands the diagnostic possibilities requiring comprehensive testing. The initial cytology results suggested tubercular lymphadenitis, so the diagnostic process became more challenging [4]. The definitive diagnosis of classical Hodgkin’s lymphoma became possible only when doctors performed an excisional biopsy together with immunohistochemical testing. Health professionals face diagnostic uncertainty during unusual lymphadenopathy cases, particularly in environments with minimal medical resources or high tuberculosis prevalence rates [5]. The early diagnosis of Hodgkin lymphoma depends heavily on tissue pathology analysis and immune staining techniques because this disease can become highly treatable when patients receive proper chemotherapy and radiation therapy. The study extends current knowledge by demonstrating why HL should be included in persistent bilateral lymphadenopathy assessment processes while validating tissue diagnosis as the definitive diagnostic method [6].

## Case presentation

A 72-year-old male presented with progressive swelling in the right cervical and left inguinal regions for six months. The swellings were painless and gradually increasing in size. There were no associated constitutional symptoms such as fever, weight loss, or night sweats. The patient denied a history of chronic cough, tuberculosis exposure, or recent infections. Family and past medical histories were unremarkable. On examination, a firm, non-tender, mobile lymph node was palpated in the right cervical region (3 × 2 cm) and left inguinal region (2.5 × 1.5 cm). No overlying skin changes, warmth, or erythema were noted. No other palpable lymphadenopathy or hepatosplenomegaly was appreciated clinically.

**Fig. 1:**
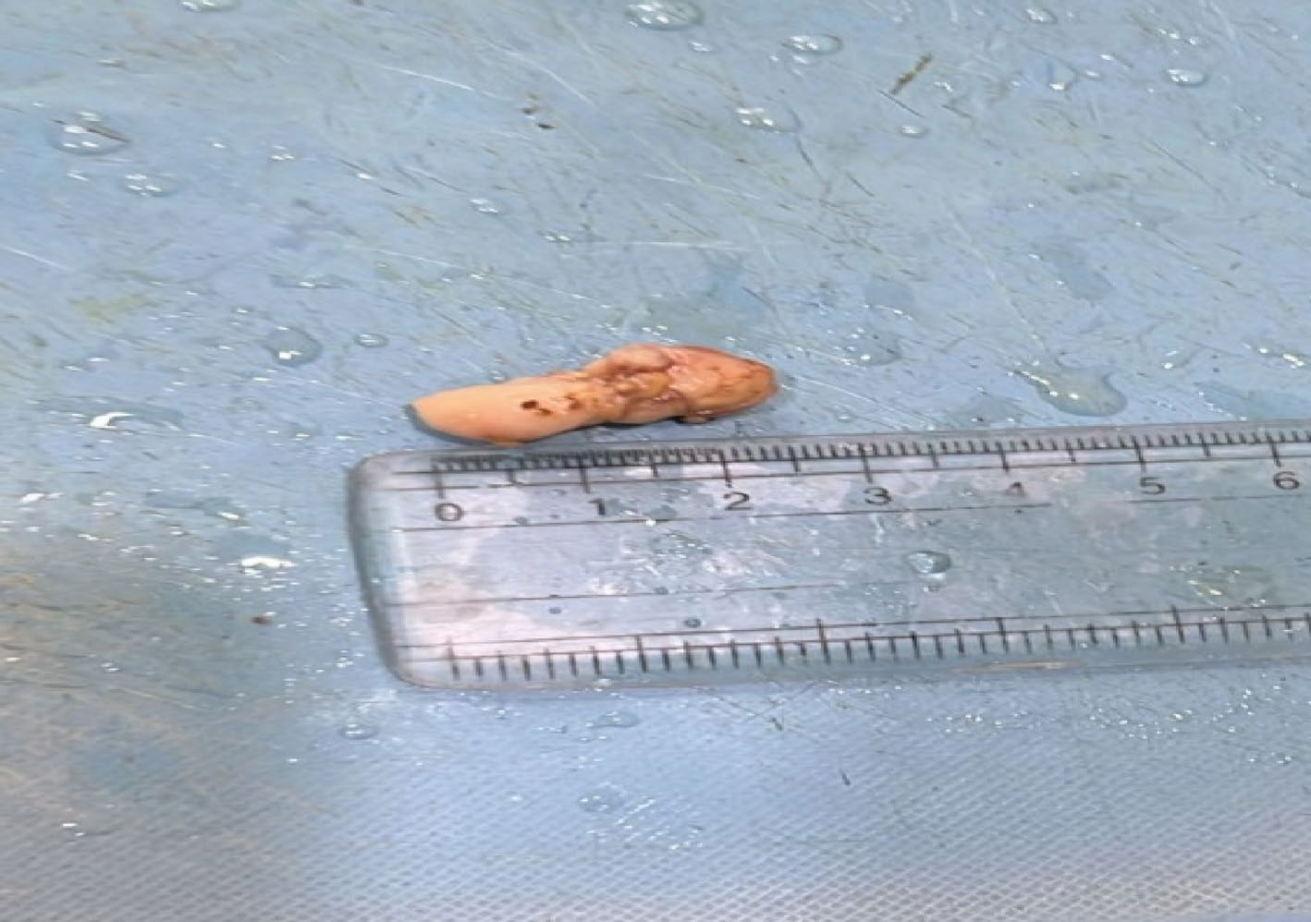
Gross appearance of excised cervical lymph node

Laboratory investigations revealed mild anemia (Hb: 10.8 g/dL) and elevated ESR (32 mm/hr), while leukocyte and platelet counts remained within normal ranges. Renal and liver function tests were unremarkable. Abdominal and pelvic ultrasonography demonstrated hepatosplenomegaly, mesenteric lymphadenopathy, and minimal ascites. Neck ultrasound additionally revealed thyroid nodules: an isoechoic right lobe lesion (TIRADS-5) and a hypoechoic left lobe lesion (TIRADS-2). Contrast-enhanced CT (CECT) showed multiple enlarged lymph nodes in the cervical, axillary, para-aortic, and mesenteric regions, the largest measuring 3.5 × 2.8 cm, along with mild pleural effusion, raising suspicion for lymphoma. Fine-needle aspiration cytology (FNAC) of the left inguinal lymph node revealed necrotizing granulomatous lymphadenitis, suggestive of tubercular lymphadenitis. However, the extensive distribution of lymphadenopathy and radiological findings raised the possibility of a malignant etiology, prompting excisional biopsy of the right cervical and left inguinal lymph nodes. Figure 1 shows greyish-white lymphoid tissue.

**Fig. 2:**
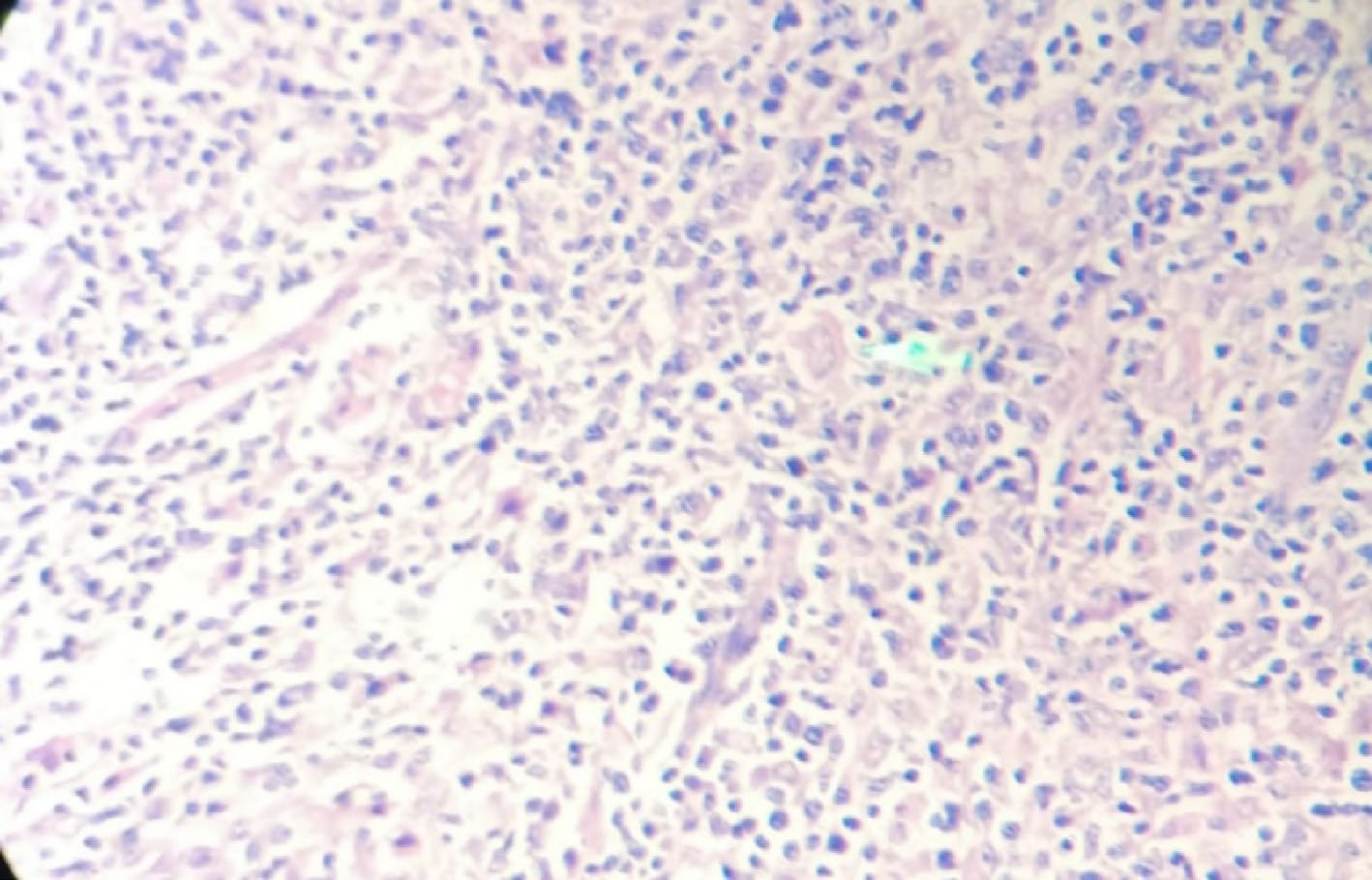
H&E microphotograph showing classic RS cells

Microscopy revealed diagnostic Reed–Sternberg cells within a polymorphous inflammatory background comprising eosinophils, plasma cells, and lymphocytes. Well-formed granulomas encircled by collagen fibers were also present, emphasizing the diagnostic challenge in differentiating HL from granulomatous conditions such as tuberculosis (Fig. 2).

**Fig. 3:**
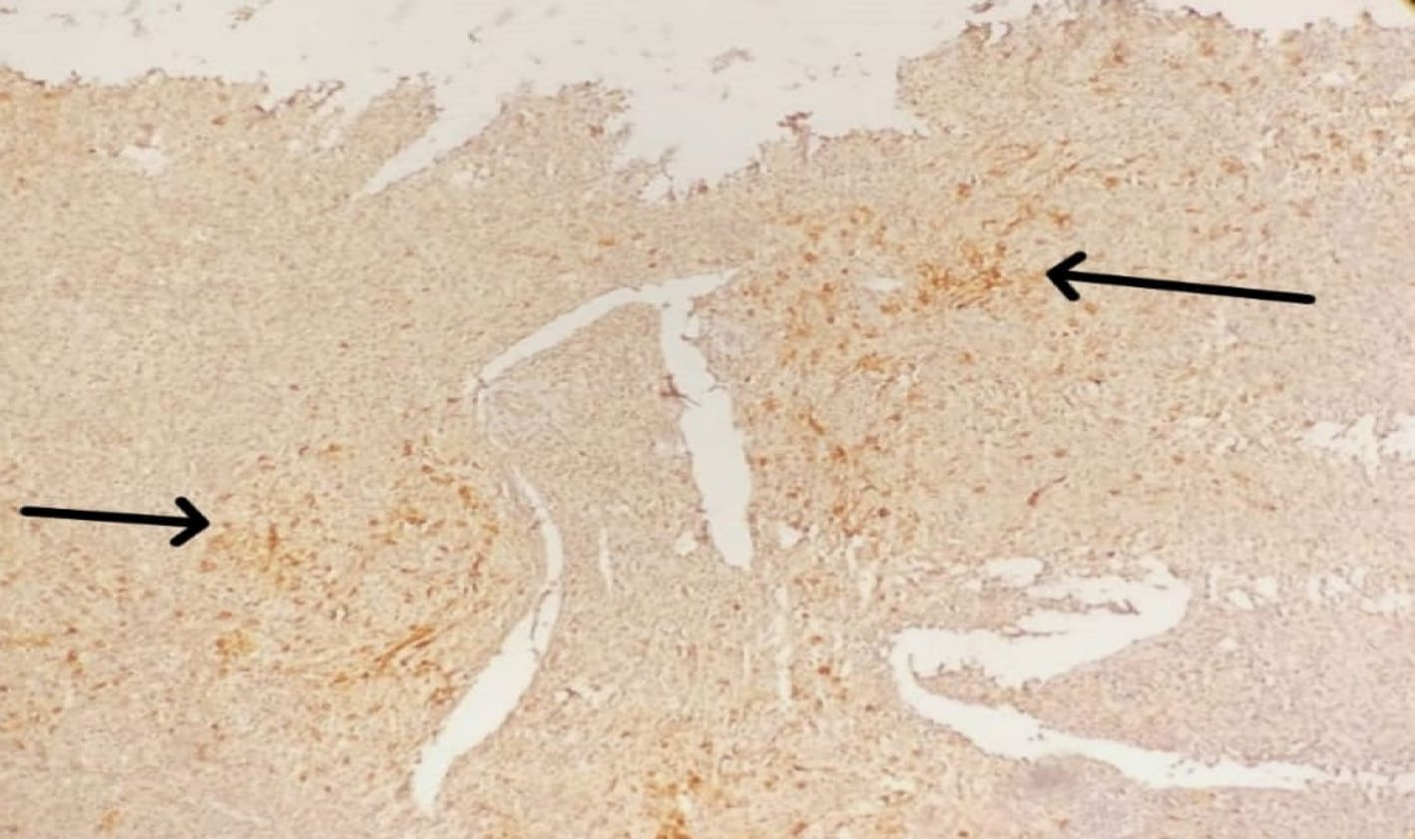
CD15 positivity in RS cells

Immunohistochemistry (IHC) was advised to confirm the diagnosis using markers CD15, CD20, CD45, and PAX-5, which are critical in verifying the presence of Hodgkin’s lymphoma. IHC studies were subsequently performed to confirm the diagnosis. Tumor cells were positive for CD15, partially positive for CD20, and negative for CD45, confirming the diagnosis of classical Hodgkin’s lymphoma (Fig. 3). Based on clinical, radiological, and histopathological findings, the patient was staged as Ann Arbor Stage II HL. He was initiated on the standard ABVD chemotherapy regimen (Adriamycin, Bleomycin, Vinblastine, and Dacarbazine). An interim PET-CT was planned after two cycles to evaluate treatment response (details awaited at the time of reporting).

The healing process after surgery progressed well without any problems emerging. Medical testing of tissue specimens after surgery validated the diagnosis. The patient displayed full compliance with both medical check-ups and prescribed medical instructions. The authors did not evaluate the chemotherapy treatment’s tolerance during their research period. The biopsy procedure resulted in no adverse events during recovery. The patient will receive future follow-up appointments to track chemotherapy development and treatment response.

### Patient perspective

The patient experienced strong anxiety after receiving the initial diagnosis because of bilateral lymphadenopathy and the suspected lymphoma. Education about Hodgkin’s lymphoma features and diagnostic processes, and treatment results calmed the patient and their family. The patient, together with their family, deeply appreciated how the healthcare team thoroughly explained procedures and provided continuous support during both excisional biopsy operations and their follow-up assessments. After the procedure took place, the patient showed contentment with the treatment he received and maintained optimism about healing and treatment results. This case highlights the diagnostic and therapeutic challenges in managing HL, particularly in elderly patients. Despite its rarity in older populations, HL should be considered in persistent, unexplained lymphadenopathy cases, especially when accompanied by suggestive histological and immunohistochemical findings. Early diagnosis and timely initiation of appropriate therapy remain pivotal in improving outcomes, even in advanced age groups. This case also underscores the importance of a multidisciplinary approach in ensuring optimal care and addressing the unique needs of elderly patients with malignancies.

## Discussion

This case underscores several diagnostic and therapeutic considerations in Hodgkin’s lymphoma (HL), particularly due to its atypical presentation with bilateral cervical and inguinal lymphadenopathy. Such a pattern of involvement is relatively uncommon and can create diagnostic uncertainty, especially in regions where tuberculosis or other granulomatous conditions are prevalent. The absence of B symptoms in this patient further complicated the clinical picture, delaying the suspicion of HL [7]. Histopathological examination of the excised lymph node demonstrated classical Reed-Sternberg (RS) cells in a fibrotic inflammatory background, confirming classical HL. Immunohistochemistry (IHC) supported the diagnosis with CD15 and PAX-5 positivity and absence of CD45. These findings differentiated classical HL from nodular lymphocyte-predominant HL, anaplastic large cell lymphoma, and diffuse large B-cell lymphoma, which share overlapping morphologic features but differ in their immunophenotypic profiles. This highlights the importance of tissue biopsy and IHC in establishing a definitive diagnosis [8]. Accurate staging is equally crucial. In this case, contrast-enhanced CT revealed involvement confined to cervical and inguinal lymph nodes, consistent with Ann Arbor Stage II disease, while bone marrow biopsy excluded marrow infiltration. Staging not only influences prognosis but also determines therapeutic strategy [9]. The patient was treated with the ABVD chemotherapy regimen, the current standard of care in HL. Although ABVD is generally effective, treatment in elderly patients carries unique challenges, including higher risks of cardiotoxicity, pulmonary toxicity, and myelosuppression. These risks necessitate vigilant monitoring, supportive care, and tailored treatment planning. Interim PET-CT scans remain a valuable tool to guide response-adapted therapy in such cases [10]. Therapeutically, CD30 expression remains of clinical relevance in HL because of the availability of brentuximab vedotin, an antibody-drug conjugate targeting CD30 that has shown benefit in relapsed/refractory settings and, more recently, in frontline treatment of advanced HL. While CD30 testing was not documented in this case, awareness of such targeted therapies is important, especially in patients with comorbidities or those who may not tolerate conventional regimens [11]. From a prognostic perspective, age is a critical factor in HL outcomes. Elderly patients often present later, experience more treatment-related toxicity, and have lower tolerance for intensive regimens compared to younger cohorts. Nonetheless, timely diagnosis and individualized management can achieve meaningful remission and improve quality of life, as illustrated in this case [12].

## Conclusion

The entire evaluation of patients exhibiting Hodgkin’s lymphoma and bilateral lymphadenopathy confirms the need for extensive medical testing of persons experiencing body-wide lymph node enlargement. Histopathology and immunohistochemistry testing offered clear diagnostic tracks to confirm this condition through disease elimination of possible infectious or granulomatous diseases. Medical professionals need to closely monitor the symptoms of lymphoma in patients demonstrating granulomatous features because these observations generate tuberculosis-like diagnostic challenges in tuberculosis-endemic zones. The correct diagnosis with suitable treatment steps emerged from professionals using early diagnostic awareness, followed by surgical biopsy procedures and a team-based medical approach. Medical professionals can use the treatment plan as a reference framework to detect Hodgkin’s lymphoma in persistent lymphadenopathy assessments they conduct when building evidence-based diagnostic methods.

## Data Availability

The data used in this study can be shared by the corresponding author upon reasonable request.
